# Composition and Diversity of Gut Bacteria Associated with the Eri Silk Moth, *Samia ricini*, (Lepidoptera: Saturniidae) as Revealed by Culture-Dependent and Metagenomics Analysis

**DOI:** 10.4014/jmb.2002.02055

**Published:** 2020-06-10

**Authors:** Kondwani MsangoSoko, Sakshi Gandotra, Rahul Kumar Chandel, Kirti Sharma, Balasubramanian Ramakrishinan, Sabtharishi Subramanian

**Affiliations:** 1 Division of Entomology, ICAR-Indian Agricultural Research Institute, New Delhi-110012, India; 2 Division of Microbiology, ICAR-Indian Agricultural Research Institute, New Delhi-110012, India

**Keywords:** Gut bacteria, localization, metagenomics, persistence, developmental stages, eri silkmoth

## Abstract

The polyphagous eri silk moth, *Samia ricini*, is associated with various symbiotic gut bacteria believed to provide several benefits to the host. The larvae of *S. ricini* were subjected to isolation of gut bacteria using culture-dependent 16S rRNA generic characterization, metagenomics analysis and qualitative enzymatic assays. Sixty culturable aerobic gut bacterial isolates comprising Firmicutes (54%) and Proteobacteria (46%); and twelve culturable facultative anaerobic bacteria comprising Proteobacteria (92%) and Firmicutes (8%) were identified inhabiting the gut of *S. ricini*. The results of metagenomics analysis revealed the presence of a diverse community of both culturable and un-culturable gut bacteria belonging to Proteobacteria (60%) and Firmicutes (20%) associated with seven orders. An analysis of the results of culturable isolation indicates that these bacterial isolates inhabited all the three compartments of the gut. Investigation on persistence of bacteria coupled with metagenomics analysis of the fifth instar suggested that bacteria persist in the gut across the different instar stages. In addition, enzymatic assays indicated that 48 and 75% of culturable aerobic, and 75% of anaerobic gut bacterial isolates had cellulolytic, lipolytic and nitrate reductase activities, thus suggesting that they may be involved in food digestion and nutritional provision to the host. These bacterial isolates may be good sources for profiling novel genes and biomolecules for biotechnological application.

## Introduction

Insect evolution has in part hinged upon their association with symbiotic microflora [1]. The gut of most insects harbors microbial symbionts whose nature varies immensely yet is crucial in influencing host biology with a considerable metabolic activities that enable insects to exploit inaccessible food sources [1, 2]. It is accepted that gut microbiota support host fitness with roles ranging from energy metabolism to shaping the immune system [3] and in the process positioning insects to perform key ecological functions in many terrestrial ecosystems [4]. With concerns over climate change and the impact of excessive use of pesticides, there is a need to reduce the environmental footprint of pest control through development of smart precision insect pest management strategies. Hence, insect-associated gut microbes have become attractive especially with regard to finding new solutions for pest control [5].

Several insect orders including Coleoptera [6], Hemiptera [7], Hymenoptera [8] and Diptera [9, 10] have been reported to harbor persistent communities of microorganisms. However, the gut microbiome of Lepidoptera is sparsely described [11]. Out of the 157,424 recognized Lepidoptera species [12], <0.1% have been screened for bacterial symbionts, revealing the limited available knowledge on bacterial associates in Lepidoptera [13]. The lepidopteran microbiome is driven by many factors ranging from the environment, diet, gut physiology, and developmental stage [13]. Studies on insect gut bacteria have utilized both cultivable and culture-independent approaches [14]. Cultivable microbes are ideal for studying symbiotic interactions sustained within the host and are also ready to be genetically manipulated for other novel applications [15]. Conventional cultivation techniques are, however, unable to characterize most microbes with 80-90% of microbial species not yet cultured. Culture- independent approaches such as using 16S metagenomics analysis provide a relatively unbiased view of the structure of complex microbial communities [16, 17]. With high-throughput sequencing approaches, several studies recently have reported abundant and diverse microbial communities in lepidopteran insect guts [18].

The eri silkmoth, *Samia ricini*, is the only species that has been completely domesticated and adopted for indoor rearing among the five commercially exploited wild silkworms in India. Eri-culture, the rearing of the eri silkmoth, has been a socio-culturally valued practice for centuries, particularly in the Brahmaputra valley of Assam and the adjoining hilly areas of Northeastern India, and is also exploited for nutrition by tribal people who use its silk to make clothing and other important biomaterials [19-21]. A study by [22] reported a diverse group of gut bacteria including *γ-proteobacteria, Firmicutes, β-proteobacteria* and *α-proteobacteria* inhabiting the gut of another wild silkmoth, *Antheraea assamensis*, belonging to the same family Saturniidae as the eri silkmoth. However, no study has been done to investigate the gut microflora of *S. ricini.* Here, we investigated the diversity of gut bacteria of *S. ricini* using a culture-dependent approach coupled with generic characterization of cultivable bacteria using 16S rRNA probes and a culture-independent approach by deploying metagenomics analysis through Next Generation Sequencing (NGS) of the V3-V4 region of 16S rRNA genes of the gut microbiome of this lepidopteran insect; the persistence of gut bacteria across developmental stages of this insect was explored using the PCR-DGGE technique. In particular, this work focused on profiling the gut microbial community within the different compartments of the gut of *S. ricini* and potentially phylogenetic relationship of predominant gut microbiota of *S. ricini* with those of other Saturniid moths found in the same environment. This knowledge would potentially enhance sustainability of commercial eri culture, for instance, through development of probiotic approaches to boost rearing of *S. ricini*.

## Materials and Methods

### Insect Culture

Eggs of eri silkworm obtained from the Central Muga Eri Research and Training Institute, Jorhat, Assam and were incubated in sterile petri-dishes under a regime of 16:8 h light and dark period with rearing temperature of 26 ± 1°C and 70% RH at the Division of Entomology, IARI, Pusa Campus; New Delhi, India, until hatching. After hatching, the first instar larvae were transferred into formalin sterilized rearing boxes and maintained on castor leaves following the standard rearing method of [23]. The first instar larvae were fed with tender leaves up to second and third instar. The fourth and fifth instar larvae were fed with mature castor leaves.

### Insect Gut Dissection and Preparation of Homogenates

Third, fourth and fifth instars of larvae and egg stages of *S. ricini* were used for extraction and isolation of gut bacteria. A total of five healthy larvae at each stage were taken and starved for 24 h prior to the extraction of guts. The larvae were rinsed in double distilled water for 30 sec followed by 70% (v/v) ethanol for 60 sec and again rinsed in double distilled water for 30 sec to remove the disinfectant. The sterilized larvae were dissected using sterile microscissors under UV-treated laminar flow to extract the gut. The extracted gut was separated into foregut (FG), midgut (MG) and hindgut (HG) and the compartments of each section were pooled into a single 1.5 ml Eppendorf tube and homogenized in 0.85% NaCl with a sterile homogenizer. The homogenized guts were briefly centrifuged to remove any undigested food material and the supernatant was used in serial dilutions and inoculum for bacterial enrichment where necessary. Newly laid eggs (less than 24 h – without disinfection by alcohol to avoid elimination of possible surface-smeared symbionts by female via egg) were used in this study; two sets of egg homogenates were prepared: Twenty eggs were collected and washed in 0.85% sterilized NaCl and designated as egg-wash (EW), and a second set was crushed in 0.85% NaCl with a sterile pestle and designated as egg-crush (EC).

### Isolation and Enumeration of Aerobic Gut Bacteria

Isolation of gut bacteria was carried out using the following culture media (all procured from HiMedia Laboratories Pvt. Ltd., Mumbai, India): Nutrient Agar (NA) media (3 g beef extract, 5 g peptone, 5 g NaCl, 20 g agar, and 1,000 ml distilled water); Tryptone Soy Agar (TSA) media (15 g casein, 5 g peptone, 5 g sodium chloride, 15 g agar and 1,000 ml distilled water) and *Pseudomonas* Isolation Agar (PIA) (10 g peptone, 10 g casein, 1.5 g dipotassium phosphate, 1.5 g magnegium sulphate, 15 g agar and 1,000 ml distilled water). The media were autoclaved at 121°C for 20 min. The gut homogenates from the various compartments were serially diluted and inoculated on agar plates in triplicate. The inoculated plates were incubated at 37°C for 24-48 h. The bacterial colonies were differentiated based on their color, size, and morphology and a single representative isolate of each morphotype was purified by streaking on corresponding agar plates repeatedly until the purity of each culture was obtained. Enumeration of the isolates was performed by calculating the number of CFUs and the values of mean colony counts were used for calculating viable counts of cultivable bacteria and expressed as CFU in 1 ml of sample. The individual purified strains were grown in Nutrient broth and maintained in glycerol stock at -80°C.

### Isolation of Facultative Anaerobic Gut Bacteria from *S. ricini*

Fifth instar *S. ricini* larvae starved for 24 h were pre-chilled for 1-2 min to immobilize them and also for ease of handling. Then, the insects were surface sterilized in 70% ethanol for 60 sec followed by rinsing in double distilled water before dissection. Individual insects were dissected as described above and immediately transferred to a 1.5 ml sterile Eppendorf tube containing sterile 0.85% NaCl solution and sealed with parafilm and kept at -20°C until next use.

### Homogenization, Serial Dilution and Gut Bacteria Culturing

Sample homogenization was carried out in an anaerobic environment comprising an air tight chamber sterilized with alcohol and continuously flashed with CO_2_. The samples were homogenized as described above and after serial dilutions, the gut homogenate was inoculated on thioglycolate medium (composed of Tryptone, 20 g/l, sodium chloride 2.5 g/l, D (+)-glucose 5.5 g/l, l-cystine 0.5 g/l, dipotassium phosphate, sodium sulphite 0.2 g/l, 1.5 g/l, methylen blue 0.002 g/l, sodium thioglycolate 0.6 g/l and agar 15 g/l) and incubated in anaerobic jars at 28°C. The number of colonies was recorded after 72 h and used to calculate the initial inoculum size. The colonies were purified by streaking on corresponding media under anaerobic conditions to obtain pure isolates.

### DNA Extraction and 16S rRNA Gene Amplification

Distinct representative purified colonies of bacteria were chosen for identification based on 16S rRNA gene probes. Individual purified bacterial isolates were grown in nutrient broth for 24 h at 37°C. After 24 h of growth, the broth cultures were centrifuged at 13,000 rpm to separate the pellet and supernatant. The supernatant was discarded and the pellet was used for DNA extraction using a modified cetyltrimethylammonium bromide (CTAB) method. The extracted DNA quality was checked on an agarose gel and quantified using a NanoDrop: 3300 FluoroSpectrometer (Thermo Scientific, USA). The 16S rRNA gene was amplified using eubacterial primers 27F-(10 μM), (5′-AGAGTTTGATCCTGGCTCAG) and 1492R-(10 μM), (5′-AAGGAGGTGATCCAGCCGCA) (Takara Bio India Pvt. Ltd.). Each reaction contained approximately 50 ng DNA, 25 μl Master Mix (2X) and 0.5 mM of each primer. The PCR was carried out in a Bio-Rad C1000-Thermal Cycler (Bio-Rad Laboratories Inc., USA) as follows: one cycle at 94°C for 5 min, 35 cycles at 94°C for 1 min, 52°C for 1 min and 72°C for 1 min 40 sec, followed by 72°C for 10 min and 4°C till next use. PCR products were examined by electrophoresis in a 1.2% agarose gel, and bands were visualized by staining with ethidium bromide. The gels were run at 100 V for1 h in TAE buffer (40 mM Tris-acetate, 1 mM ethylene-diamine-tetra-acetic acid (EDTA); pH 7.4). Gels were visualized under UV in the Gel Documentation system of Alpha ImagerTM gel imaging system (Alpha Innotech, USA). For analysis of the persistence of gut bacteria through PCR denaturing gradient gel electrophoresis (DGGE) – total genomic DNA extracted from overnight grown homogenates from egg, foregut, midgut and hindgut compartments was amplified using F984GC (5′-AACGCGAAGAACCTTAC) and R1378 (5′-CGGTGTGTACAAGGCCCGGGAACG) as the forward and reverse primers, respectively, with a GC clump (5′-GCCCCGGGGCGCGCCCCGGGCGGGGC GGGGGCACGGGGGGAACGCGAAGAACCTTAC) attached to the 5' end of primer F984GC (Takara Bio India Pvt. Ltd.) using the PCR conditions as described earlier. The amplicons were checked by electrophoresis on 1.2% gel followed by purification for DGGE analysis using the ChargeSwitch Pro PC Cleanup Kit (Invitrogen, USA). About 200 ng of purified PCR amplicons was used for the DGGE. Briefly, the purified amplicons were electrophoresed with a linear denaturing gradient of 30%-70% denaturant on a Detection Code System (Bio-Rad) with an initial pre-run for 2 h at 120V (before sample loading), followed by 70V for 15 min (58°C) and a final constant voltage of 38V for 15 h. DGGE band profiles were visually identified and scored as present or absent. Bacterial colony bands in the gel appearing in the same position were regarded as the same genotypes. Sanger’s sequencing of the 16S rRNA gene PCR amplicons was outsourced using the same primers as indicated above (AgriGenome Labs Pvt. Ltd., India). Only trimmed high-quality partial 16S rRNA sequences were checked with their closed relatives using the NCBI-BLAST algorithm and bacterial isolates were identified based on their similarity with existing sequences.

### Diversity and Distribution of Culturable Aerobic Gut Microbes

To determine the diversity and distribution of gut microbes across different gut sections (foregut, midgut and hindgut); the identified gut bacteria isolated from the above mentioned sections were analyzed with *Shannon– Wiener index (H’)*. The data on the gut bacteria of the eri silkworm isolated from different sections of the gut across life stages were subjected to diversity analysis. The Shannon–Wiener and Simpson diversity indices were computed as described by [24].

**Shannon–Wiener index (H’).** The Shannon–Wiener index [25] is represented as:

H'=−∑Pi(lnPi)

Where *Pi = n/N*, n is the number of individuals of a species in the sample, *N* is the total number of individuals of all species in the sample and *ln* is the natural logarithm. A value near zero indicates that the sample is dominated by a single species, whereas a value near 4.5 indicates that the number of individuals is evenly distributed amongst all the species present.

### Phylogenetic Analysis

To assess the phylogenetic relationships of the gut bacteria isolates from eri silkmoth, and to compare dominant gut bacteria between eri silkmoth and Muga silkworm as these are closely related insects in the family Saturniidae, the 16S rRNA sequences of gut bacterial isolates from both these insects were assembled and aligned using BioEdit Sequence Alignment Editor V. 7.0.5.3 [26] and MEGA 7.0 [27]. A phylogenetic tree was constructed by the neighbor-joining method with Kimura two-parameter correction [28]. To calculate the support for each clade, bootstrap analysis was performed with 1,000 replications [29].

### 16S rRNA Amplicon-Based Illumina Library Preparation

The fifth instar eri silkworm larvae were used for metagenomics analysis of bacteria and five healthy larvae of approximately the same size were used. Insect dissection was carried out as described earlier. Total genomic DNA to be used for metagenomics analysis was pooled by direct extraction of DNA from the midgut and hindgut (to cutter for both cultivable and uncultivable bacteria) and from 24 h nutrient broth-enriched growth using homogenates from the respective gut compartments (to enrich cultivable bacteria) using a DNeasy Power Kit (Qiagen, USA). The DNA was later pooled and quantified using a Qubit Fluorimeter (V.3.0). TheV3-V4 region of 16S rRNA was ampliﬁed using speciﬁc V3 Forward primer- (5′-CCTACGGGNBGCASCAG) and V4 Reverse primer- (5′-GACTACNVGGGTATCTAATCC). The ampliﬁed product was checked on 2% agarose gel and gel puriﬁcation was done to remove non-speciﬁc ampliﬁcations. In addition, 5 ng of ampliﬁed product was used for library preparation using the NEBNext Ultra DNA Library Preparation Kit (New England Biolabs Inc., USA). The library quantiﬁcation and quality estimation were done in Agilent 2200 TapeStation. The prepared library was sequenced in Illumina HiSeq 2500 with 2*250 cycles chemistry.

### Bioinformatics Analysis Pipeline

Briefly, the following analysis was performed: Fastq quality checking was done to check base quality, base composition and GC content. This was followed by filtering and identification of the V3-V4 region from paired- end data through read trimming and identification of V3-V4 sequences and construction of consensus sequence from paired-end reads. After this, operational taxonomic unit (OTUs), taxonomy classiﬁcation and relative abundance were carried out by identification of OTUs, assigning of taxonomy to the identified OTUs and identification of reads and OTU abundance.

### Metagenomics Analysis of the V3-V4 Region

The raw reads obtained from Illumina sequencing platform after demultiplexing were subjected to FastQC program (ver. 0.11.8) to check the quality of the reads with default parameters. The base quality (Phred Score; Q), base composition, GC content, ambigious bases (other than A, T, C, and G) and adapter dimers were thoroughly checked prior to the bioinformatics analysis.

### Identiﬁcation of V3-V4 Region from Paired-End Reads

The following steps were performed to extract the V3-V4 region from Illumina paired-end sequences. In brief, the identification of the V3-V4 region from paired-end reads involved trimming of sequencing primers by using specific forward V3 and reverse V4 primers using In-house PERLscript and only properly paired-end reads with Phred score quality (Q>20) were considered for the V3-V4 consensus generation. This was followed by building a consensus V3-V4 region from trimmed paired-end reads by using the primer-trimmed, high quality paired-end reads by merging them to get amplicon consensus FASTA sequences using FLASH program (Ver. 1.2.11) with minimum overlap of 10 bp to maximum overlap of 240 bp with zero mismatches. All the consensus reads were formed with an average contig length of 350 to 450 bp. Before the start of the analysis, chimeric sequences were removed using the de novo chimera removal method UCHIME (version 11).

### Reads Processing and Taxonomy Analysis

The Operational Taxonomic Units (OTU) picking and taxonomy classiﬁcation were performed using the pre- processed consensus V3-V4 sequences. Pre-processed reads from all samples were pooled and clustered into OTUs based on their sequence similarity using Uclust program (similarity cutoff = 0.97) available in QIIME software (QIIME1 software Version: 1.9.1). The representative sequences from each of the clustered OTUs were picked and aligned against SILVA database sequences using PyNAST program. Taxa other than the top 10 were categorized as others and sequences that did not have any alignment against taxonomic database were categorized as unknown. The total sum normalization (TSS) approach, which normalizes count data by dividing featured OTUs by the total number of OTUs in each sample, was used to modify the OTU table into relative abundance plot.

### Alpha Diversity and Rarefaction Curves

The microbial diversity within the samples was analyzed by calculating Shannon (measure observed OTUs abundance and accounted for both richness and evenness), Chao1 (estimates species richness) and observed species metrics (QIIME software).

### Functional Characterization of Culturable Gut Bacterial Isolates

**Cellulase assay.** The gut bacterial isolates were screened in order to establish their ability to utilize cellulose. In short, individual bacterial isolates were inoculated on Carboxyl Methyl Cellulose (CMC) agar plates and incubated at 37°C for 48 h. CMC degradation was checked by flooding the plates with 0.2% Congo red solution for 30 min [30]. The degradation of CMC was indicated by a yellow zone around the colony. Enzyme activity was determined as the diameter of the colony plus the surrounding clear zone divided by the diameter of the colony [31].

**Lipase assay.** The gut bacterial isolates were screened for lipolytic activity by using Rhodamine B medium and plates were incubated at 37°C for 48 h. The degradation of the substrate causes the formation of orange to pink zones around bacterial colonies. Enzyme activity was determined by measuring the diameter as above [32].

### Nitrate Reductase Assay for Facultative Anaerobic Gut Bacteria

The anaerobically isolated gut bacteria were assayed to establish if they may also be involved in nitrate reduction following a protocol for qualitative nitrate reductase assay adopted from Sigma (Sigma-Aldrich.com). In brief, the anaerobic bacterial isolates were inoculated in nitrate broth (peptone 5 g/l, meat extract 3 g/l, potassium nitrate 1 g/l - final pH 7.0). The samples were incubated at 37°C for 24 h. After incubation, five drops of sulfanilic acid and α-Naphthylamine solution (dissolved in 5N acetic acid) were added to the tubes containing the cultures and shaken thoroughly. A distinct red or pink color development after a few minutes was an indication of nitrate reduction.

## Results

### Enumeration, Isolation and Identification of Aerobic Gut Bacteria

**Fig. 1:**
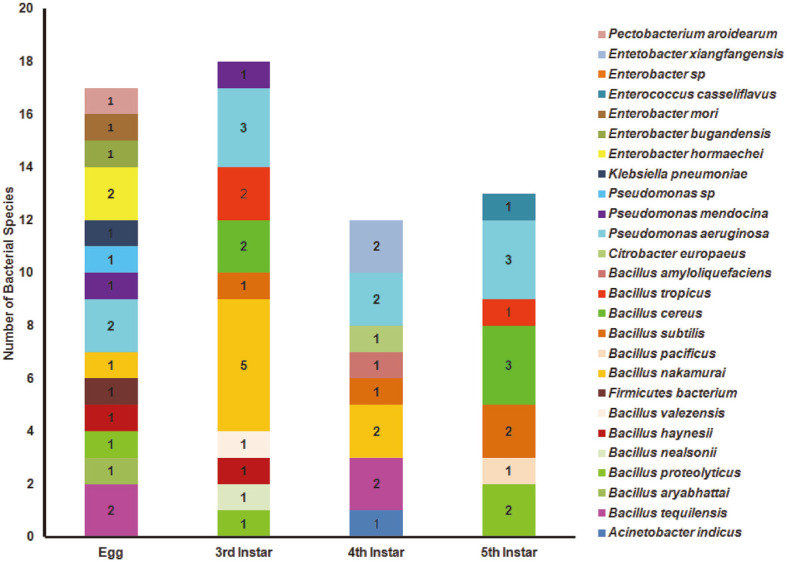
Overview of the number of bacterial isolates inhabiting the egg, third, fourth, and fifth instar stages of *S. ricini*. Different colors represent the different gut bacterial isolates isolated from each stage as identified by 16S rRNA gene sequences.

The gut bacteria were profiled across different gut compartments (foregut, midgut and hindgut) and across selected developmental stages (egg, third, fourth and fifth instar) of *S. ricini*. The results of gut bacterial isolation and enumeration from the different compartments of the intestinal tract and across developmental stages are presented in Fig. 1 and Table S1. No significant differences were found in colony-forming units (CFUs) among the three media types and gut compartments or across instars except for the fifth-instar stage in which the midgut section had significantly (*p* = 0.0464) higher CFU compared with the foregut and hindgut. However, generally, the midgut of *S. ricini* had the highest CFU count compared with the foregut and hindgut compartments for the other screened stages. Generic identification of these isolates was carried out using PCR amplification of the 16S rRNA gene sequencing. A comparative analysis of the resulting 60 sequences was carried out with the closest relatives in the National Center for Biotechnology Information (NCBI) database. Similarity checks with GenBank showed 58 isolates with similarity ≥ 97% to their closest relatives from the NCBI database. Two isolates, *Pseudomonas* sp. ERI056-EW-IND and a *Firmicutes bacterium* isolate ERI070-EC-IND, had similarities of 96% and 85% with their closest relatives in the NCBI database possibly suggesting that they may be a different species and genus altogether. The 16S rRNA gene sequences of the isolates were submitted to GenBank and assigned accession numbers as provided in Table S2.

### Enumeration and Isolation of Facultative Anaerobic Gut Bacteria

The anaerobic gut bacteria were profiled across different gut compartments (foregut, midgut and hindgut). The results of gut bacterial isolation and enumeration from the different compartments are provided in Table S3. No significant differences were found in colony forming units (CFUs) among the three gut compartments, though the hindgut had numerically the highest CFU value compared to the other two gut compartments. In total, 12 anaerobic bacterial isolates were isolated from *S. ricini* across the three gut compartments with the majority being identified from the midgut and hindgut. Similarity checks with GenBank showed almost all of them had similarity ≥ 97% to their closest relatives from the NCBI database except for isolates ES-ANE-EFG-4 and ES-ANE-EMG-5-A. The 16S rRNA gene sequences of the isolates were submitted to GenBank and assigned accession numbers as detailed in Table S4.

### Diversity of Culturable Aerobic Gut Bacteria from *S. ricini*

The diversity of the culturable aerobic gut bacteria was estimated by the Shannon-Weiner index. This index was calculated based on the total number of bacterial isolates characterized in each compartment of the gut as it is an important tool in deciphering the dominance of species in a particular community structure. The data on diversity indices from the different sections of the gut of *S. ricini* are given in Table S5. No significant differences in Shannon-Wiener index was found between the gut compartments that were sampled. However, the midgut was relatively more even and had higher species richness (14 species) compared with the hindgut (nine species) and foregut (seven species).

### Denaturing Gradient Gel Electrophoresis

**Fig. 2:**
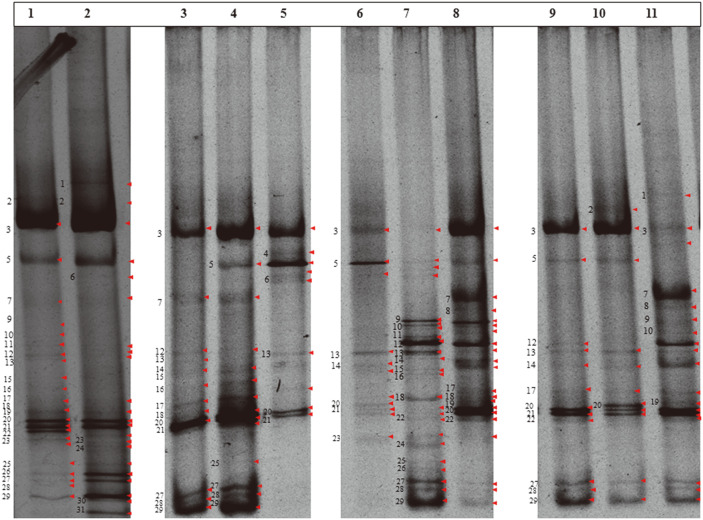
The above image is a denaturing gradient gel electrophoresis (DGGE) of amplified partial 16S rRNA gene at the respective developmental stages, namely egg, third, fourth and fifth instars of *S. ricini*, to show persistence of gut bacteria across stages. Key: 1=Egg wash, 2=Egg crush, 3=Foregut-3^rd^ Instar, 4=Foregut-4^th^ Instar, 5=Foregut-5^th^ Instar, 6=Midgut-3^rd^ Instar, 7=Midgut-4^th^ Instar, 8=Midgut-5^th^ Instar, 9=Hindgut-3^rd^ Instar, 10=Hindgut-4^th^ Instar, 11=Hindgut-5^th^ Instar for Eri silkworm, *S. ricini*. [As this figure is a consolidated image of two gels, the original gel profiles are given in Figs. S1 and S2.

A total of 31 amplified bacterial bands were detected in the DGGE profile of eggs. After scrutiny of the DGGE bands with respect to other developmental stages (third, fourth and fifth instars) of *S. ricini*, it was observed that 61, 81, 68% of the initial amplified bacterial bands as seen in egg stage were maintained at the same position with the DGGE profiles of the third, fourth and fifth instars, respectively. The results of the amplified profiles of the partial 16S rRNA bacterial isolates by DGGE are shown in Fig. 2. The full-length DGGE profiles are given in Figs. S1 and S2.

### 16S V3-V4 Region Metagenomic Analysis

To explore microbial diversity of both cultivable and uncultivable gut bacteria associated with *S. ricini*, a 16S rRNA V3-V4 metagenomic sequencing analysis of the midgut and hindgut was conducted using fifth instars. A total of 6,998 OTUs were identified from 365,963 reads. From 6,998 total OTUs, 6,381 OTUs with less than 5 reads were removed and 617 OTUs were selected for further analysis. A graph depicting relative reads and OTU proportion is provided in Fig. S3.

### Microbial Diversity

**Fig. 3:**
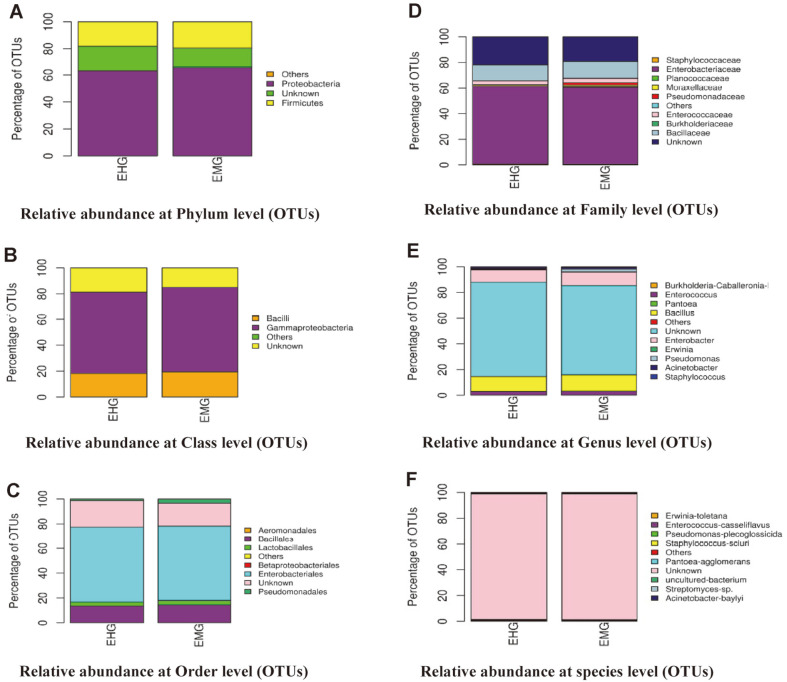
Relative abundance plot at phylum (A), class (B), order (C), family (D), genus (E), and species (F) level based on OTUs. The different colors depict proportionate distribution of each group at the different taxonomic levels. Key: EMG-Eri Midgut; EHG-Eri Hindgut.

A total of 617 Operational Taxonomic Units (OTUs) belonging to two phyla and seven associated orders of bacteria were observed in our samples. The most frequently observed phylum that was dominant in both the midgut (65.5%) and hindgut (63.14%) was the Proteobacteria followed by Firmicutes –midgut (19.4 %) and hindgut (18.17%). All the other phyla had an abundance of less than 1%, in both gut compartments, and 15% (midgut) and 18.7% (hindgut) of OTUs were unassigned. The most abundant orders were Enterobacteriales (60.1%-midgut and 60.5%-hindgut), Bacillales (14.3%-midgut and 13.4%-hindgut) and Pseudomonadales (3.3%-midgut and 1.2%-hindgut). The relative abundance plot at phylum, class, order, family, genus and species level based on OTU is shown in Fig. 3 and a detailed sample-wise OTU table is given in Suppl. File1.

### Alpha Diversity

**Fig. 4:**
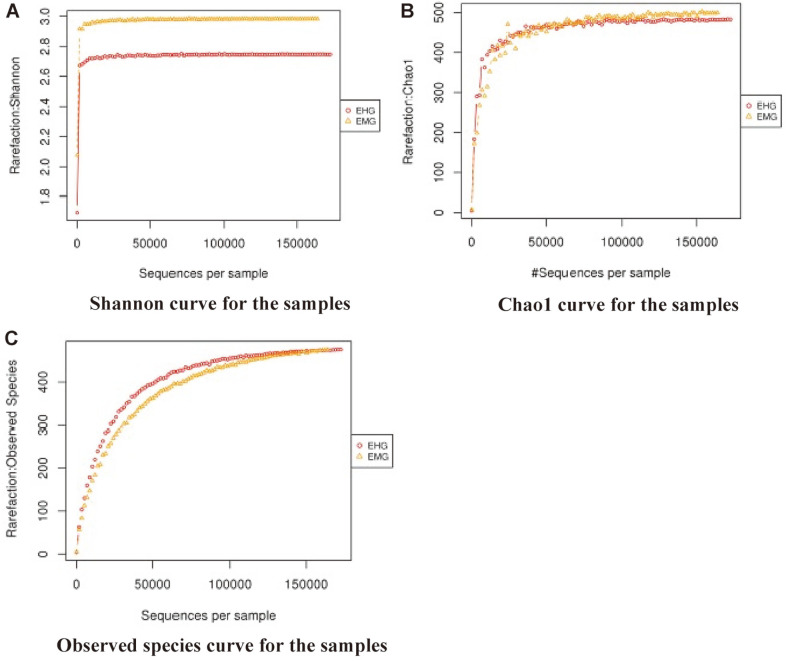
Shannon (A), Chao1 (B) and Observed species (C) metrices showing species diversity, richness and count of unique OTUs identified from the midgut and hindgut of *S. ricini*.

The microbial diversity within the samples was analyzed by calculating Shannon, Chao1 and observed species metrics, and the results of these indices are depicted in Fig. 4. The results of the Shannon metric indicated that the midgut compartment had more species richness and evenness than the hindgut compartment. Similar observations were found with Chao1 which estimates species richness only whereas in terms of unique OTUs, the hindgut compartment appeared to have more unique OTUs than the midgut.

### Cellulolytic and Lipolytic Screening of Aerobic Gut Bacterial Isolates

The sixty bacterial cultures isolated from *S. ricini* were subjected to qualitative screening for presence of cellulolytic activity. Amongst the 60 isolates screened isolates for cellulase activity, 30 and 44 bacterial isolates were found to possess cellulolytic and lipolytic activity, respectively. The cellulolytic index ranged from 1.13 to 2.84 while the lipolytic index ranged from 1.13 to 3.13. The results of cellulolytic and lipolytic indices for all the bacterial isolates are provided in (Table S6).

### Screening for Nitrate Reductase of Facultative Anaerobic Gut Bacterial Isolates

The anaerobic gut bacterial cultures were assayed for presence of nitrate reductase activity and the results indicated that all the bacterial isolates had nitrate reductase ability except for isolates ES-ANE-EMG-5, ES-ANE- EMG-5-A and ES-ANE-EHG-4 (Table S7).

### Phylogenetic Analysis of Culturable Aerobic and Facultative Anaerobic Gut Bacterial Isolates

**Fig. 5:**
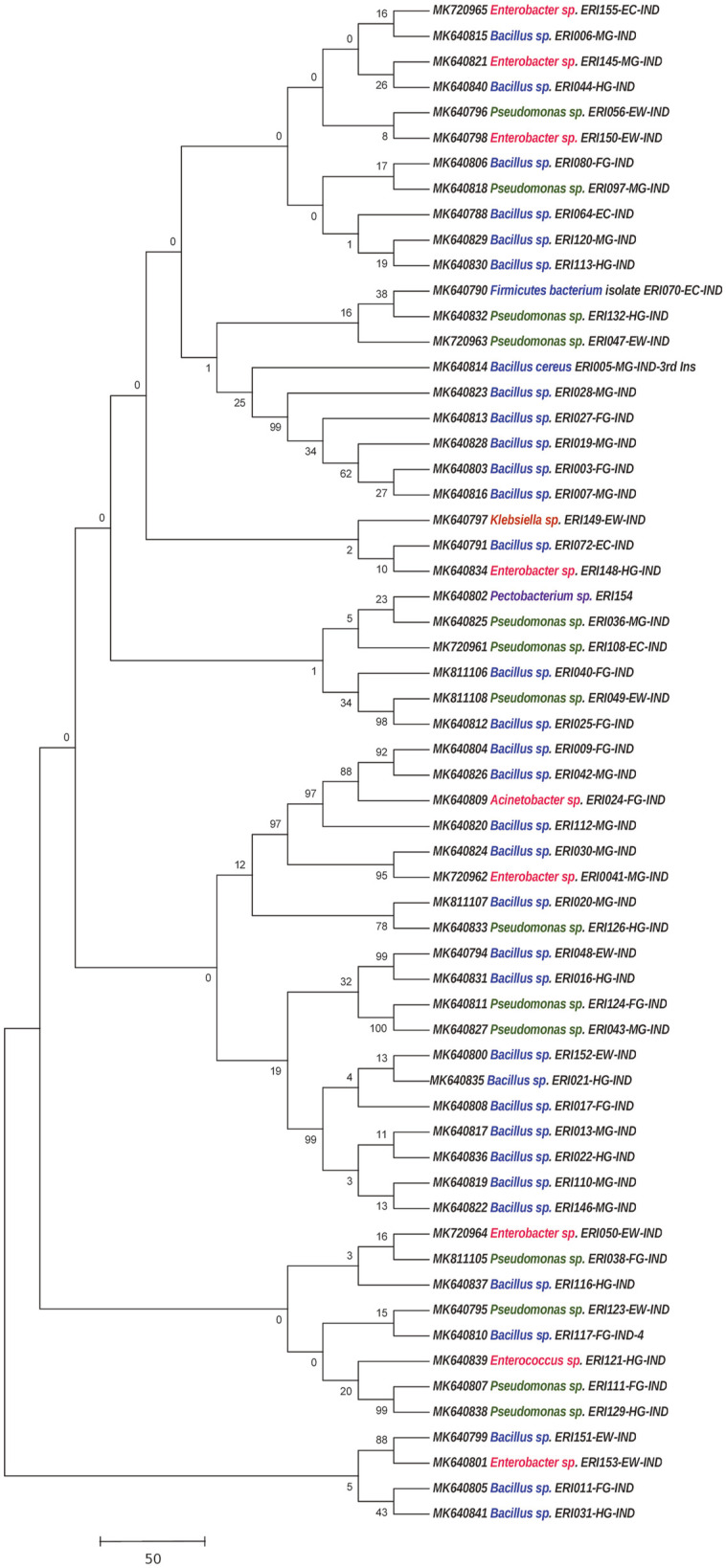
Phylogenetic tree showing evolutionary relationships of gut bacteria isolated from *S. ricini*. The 16S rRNA gene sequences were aligned using BioEdit 7.0, ClustalW programs. The percentage of replicate trees in which the associated taxa clustered together in the bootstrap test (1,000 replicates) are shown next to the branches. The tree is drawn to scale, with branch lengths (next to the branches) in the same units as those of the evolutionary distances used to infer the phylogenetic tree. The phylogenetic tree was constructed based on the neighbor-joining algorithm with Kimura two- parameter corrections, using the MEGA 7.0.

Phylogenetic relationship analyses of culturable aerobic and facultative anaerobic gut bacteria 16S rRNA gene sequences from *S. ricini* showed Firmicutes as the dominant phyla with thirty-six bacterial isolates for aerobic bacteria (Fig. 5) and a single isolate, ES-ANE-EFG-6, for facultative anaerobes (Fig. S4). The Proteobacteria formed the second dominant phyla comprising the thirteen bacterial isolates for aerobic culturable gut bacteria and eleven bacterial isolates for anaerobic gut bacteria isolates.

## Discussion

Insects establish symbiotic microbial associations through vertical transmissions or diet and these symbionts may be involved in cellular digestion of food via nitrogen fixation, cellulose digestion, and host nutrition support among others [33]. The results of our study suggest that the majority of culturable aerobic and facultative anaerobic gut bacteria associated with *S. ricini* belong to Firmicutes and Proteobacteria. However, metagenomics analysis revealed that the gut of *S. ricini* is very complex and is colonized with both culturable and uncultivable bacteria spanning two phyla (Proteobacteria and Firmicutes) over seven orders and ten genera. The OTUs analysis at the phylum level suggested that most of the gut bacteria in the gut of *S. ricini* belonged to Proteobacteria (60%) followed by Firmicutes (20%). A study by [34] reported similar results that Proteobacteria and Firmicutes as the dominant phyla in insect gut samples were represented by 57.4% and 21.7% of the sequences, respectively. Similarly, our findings indicated that the bacteria belonging to the order Entrobacteriales (60%) dominated the gut bacteria of *S. ricini* followed by Bacillales (15%). The majority of the aerobic and facultative anaerobic taxonomic units at phylum, class and order level identified through cultivable techniques were also confirmed through metagenomics analysis; however, metagenomics sequencing results suggest that the gut of *S. ricini* is associated with a more complex gut bacteriome than that revealed through cultivable approach. This was observed from the results of the culture-dependent approach which revealed gut bacteria belonging to three orders (Bacillales, Enterobacteriales and Pseudomonadales), whereas metagenomics results revealed two folds of that observed with culturing alone. As reported in obligate insect symbionts, most microbes that are tightly associated with their hosts have highly reduced genomes and only retain essential complementary gene sets for encoding key biosynthetic pathways required by their hosts and as such cannot be cultured on conventional media [35, 36]. This may be the case with the gut of *S. ricini* as the majority of the taxonomic units unraveled with metagenomics analysis could not be cultured on common bacteriological media. It should also be pointed that about 15% (midgut) and 18.7% (hindgut) of phyla could not be assigned. It is possible that these could be rare or novel microbes not classified before. However, this may also be attributed to resolution failure as we only targeted the V3-V4 region of the 16S rRNA as reported by [37], who indicated that resolving the taxonomy based on the V4 region could be a challenge as sequences tend to be near identical to several other sequences in the database because they may accrue larger proportions of reads at the higher taxonomic tree.

The eri silkmoth is polyphagous on different plant hosts which possess relatively high cellulose, hemicellulose and lignin [38]. It is apparent from our findings and those reported by other authors in other lepidopteran insects that Proteobacteria and Firmicutes dominate gut bacteria symbionts across insect orders and as such may be contributing greatly to host physiology. An earlier study had demonstrated that gut bacteria such as *Enterobacter* and *Enterococcus* isolated from a lepidopteran insect aid the host with moderation of gut pH and enhance the alkalinity which plays a critical role in tannin digestion [39]. Another study by [40] had earlier reported that caterpillars lack a resident gut microbiome suggesting reasons such as their gut morphology and rapid digestion throughput which may not allow microbes to persist in their gut. Using *S. ricini* as our study system and also considering that this insect is phytophagous, we isolated culturable bacteria across the different developmental stages starting from egg through third, fourth and fifth instars with the assumption that some bacterial isolates would be passed on from the egg and persist in the later stages of development. Our results suggest that the egg surface of *S. ricini* appears to harbor an inherited bacterial community as revealed by egg extraction and egg wash homogenates. Most gut bacteria live extracellularly; and [3] reported that heteropteran insect gut symbionts are added to the egg surface by females in specialized secretion to be later acquired by the hatching neonates. This may also be a possible route of lepidopteran neonate larvae to acquire these bacteria as they have been reported to gnaw their egg shell while hatching and ingesting it afterwards [13]. Our observations are in agreement with the already published reports [41-42] that a number of microbes including *Enterococcaceae* and *Lactobacilli* were stable across the different growth stages of *S. littoralis* and *H. armigera*. A study by [43] also indicated that insect guts appear to have a core community of gut bacterial species that will persist in their gut irrespective of diet and other factors.

The results of DGGE from our study suggest that some bacterial isolates persist in the gut across the different growth stages as > 60% of the bacterial bands amplified at egg stage maintained the same positions in the gel at the third, fourth and fifth instar, respectively, as depicted in Fig. S5. Moreover, the metagenomics analysis performed on the fifth instar, which is the last stage before pupariation, revealed the presence of different microbial groups, thus, we can presume that these had persisted in the gut and may be part of a core community of bacterial symbionts associated with this insect.

The phylogenetic analysis of the aerobic gut bacterial 16S rRNA gene sequences showed that bacterial isolates belonging to the same phyla/genus with high cluster stability suggests that they share a close relationship. Some bacterial isolates that were identified at the egg stage had a very close phylogenetic relationship with later identified isolates suggesting that these could probably be the essential symbionts that may be passed on via egg smear by the female adults and may be crucial for the physiology of the larvae. An attempt was made to compare the dominant gut bacterial genera between *S. ricini* and *A. assamensis* [19] and the results of phylogenetic relationships between these two insects showed that the dominant bacteria formed three major clades (Fig. S6). Six isolates from *S. ricini* together with seven from *A. assamensis* comprised clade I, while three isolates from both *S. ricini* and *A. assamensis* formed three different sub-branches within clade I suggesting that they are closely related. The second clade (clade II) comprised 12 isolates from *S. ricini* and 4 from *A. assamensis.* The third clade (clade III) comprised 11 isolates from *S. ricini* and four from *A. assamensis.* The gut bacterial isolate KJ672335 from *A. assamnesis* formed a sub-clade with the nine bacterial isolates of *S. ricini* suggesting a close relationship between these isolates. A single isolate from *S. ricini* bearing accession number: MK640791 formed a separate branch from clad III suggesting that this bacterial isolate may be distantly related to the other isolates.

Qualitative screening of the bacterial isolates from *S. ricini* revealed that these culturable gut bacteria possess cellulolytic, lipolytic and nitrate reductase activity. As such we presume that these gut bacteria may be important symbionts contributing to food digestion in *S. ricini.* The eri silkworm is a domesticated insect and is reared throughout the year. Therefore, the knowledge on the presence of beneficial gut bacteria with cellulolytic and lipolytic activities offers the possibility of development of probiotics to enhance commercial eri rearing. In addition, this insect could also be a good resource for bio-prospecting of novel microbes for commercial production of cellulase and lipase enzymes for industrial applications. Insects throughout their evolution have established associations with different bacteria as these bacterial symbionts perform multiple physiological functions such as degradation of complex hemicellulose components into simple forms in the insect gut, thus facilitating better insect nutrition [43, 44].

This study was aimed at exploring gut bacteria associated with the eri silk worm. Utilizing culture-dependent techniques and PCR amplification of the 16S rRNA gene sequencing, our study identified a number of culturable bacteria colonizing the gut of *S. ricini* including *Bacillus,* spp*., Citrobacter* sp., *Enterobacter* sp., and *Pseudomonas* spp*.* The results of metagenomics sequencing reconfirmed the presence of the taxonomic units obtained through culturing and revealed additional gut bacterial orders not identified during culturing (both culturable and unculturable) including Aeromonadales, Lactobacillales and Betaproteobacteriales. Some of these may be transient bacteria; however, the results of our study suggest that some of the associated gut bacteria may probably be core symbionts of this insect. Further studies are required to throw more light on their symbiotic status as some of the bacterial isolates were found to possess cellulolytic, lipolytic and nitrate reductase activities and may be good sources for profiling novel genes and biomolecules for industrial use.

## Supplemental Materials



Supplementary data for this paper are available on-line only at http://jmb.or.kr.
