# Supplementary material for: Composition and Diversity of Gut Bacteria Associated with the Eri Silk Moth, Samia ricini, (Lepidoptera: Saturniidae) as Revealed by Culture-Dependent and Metagenomics Analysis
Source: J Microbiol Biotechnol. 2020 Jun 10;30(9):1367–78. doi: 10.4014/jmb.2002.02055 (PMC9728191; doi:10.4014/jmb.2002.02055)
Supplement: Supplementary file 1 [file JMB-30-9-1367-supple.pdf]

J. Microbiol. Biotechnol.
https://doi.org/10.4014/jmb.2002.02055

**Supplemental File – JMB submission**

**Composition and diversity of gut bacteria associated with the eri silk moth, *Samia ricini*, (Lepidoptera: Saturniidae) as revealed by culture-dependent and metagenomics analysis**

**Corresponding author:** Subramanian Sabtharishi

**Supplementary Table S1.** Mean colony-forming units (CFU per ml ± SE) of bacteria isolated across growth stages and gut compartments of *S. ricini*

| Egg-crush | | Egg-wash | | 3rd-instar | | 4th -instar | | 5th-instar | |
|---|---|---|---|---|---|---|---|---|---|
| Media | Log CFU/ml (x $10^8$) | Media | Log CFU/ml (x $10^8$) | Section | Log CFU/ml (x $10^8$) | Section | Log CFU/ml (x $10^8$) | Section | Log CFU/ml (x $10^8$) |
| PIA | 7.57±1.38 | PIA | 8.61±0.71 | FG | 9.23±0.63 | FG | 9.00±0.50 | FG | 7.09±0.58 |
| NA | 8.25±1.27 | NA | 8.50±0.75 | MG | 9.84±0.51 | MG | 9.22±0.53 | MG | 8.42±0.54 |
| TSA | 9.85±1.26 | TSA | 9.38±0.72 | HG | 8.46±1.04 | HG | 8.76±0.50 | HG | 8.21±0.77 |
| Mean | 8.5500 | | 8.8300 | | 9.1800 | | 8.9900 | | 7.9100 |
| p-Value | 0.4883 | | 0.5149 | | 0.3442 | | 0.7645 | | 0.0464 |
| SE(d) | 1.8430 | | 1.0300 | | 0.9390 | | 0.6200 | | 0.5350 |
| Tukey HSD at 5% | NS | | NS | | NS | | NS | | 1.3430 |

SE (d), Standard error of difference (with three replicates). Mean CFU values were calculated from the gut bacteria of egg, 3rd, 4th and 5th -instar larvae of *S. ricini* at a dilution factor of x$10^8$. Analysis of variance, SE and Tukey's honest significant difference (HSD) test (α =0.05) were calculated using SAS. CFU=Colony Forming Unit, PIA=*Pseudomonas* Isolation Agar, NA=Nutrient Agar, TSA=Tryptone Soy Agar, FG=Foregut, MG=Midgut, HG=Hindgut.
Key: EC= egg crush, EW= egg wash, FG= foregut, MG= midgut, HG= hindgut

**Supplementary Table S2.** Microbial abundance in different gut compartments of *S. ricini.* Data are presented as $\log_{10}$ values of CFU (log CFU) ± standard error of the mean (SE).

| Gut Compartment | Bacterial Count (log CFU /mL) |
|---|---|
| Foregut | 9.39 ± 1.38 |
| Midgut | 9.42 ± 1.39 |
| Hindgut | 10.6 ± 1.34 |
| Mean | 9.81 |
| SE(d) | 1.938 |
| P-value | 0.7835 |

**Supplementary Table S3.** Gut bacteria isolates from the different compartments and selected growth stages of *S. ricini* identified by 16S rRNA gene sequence analyses

| Isolate ID | Accession Number | Species | Stage | Organism with closest match from GenBank | Similarity (%) |
|---|---|---|---|---|---|
| ERI064-EC-IND | MK640788 | *Bacillus* sp. ERI064-EC-IND | Egg | *Bacillus tequilensis* strain JAAKPT | 99 |
| ERI155-EC-IND | MK720965 | *Enterobacter* sp. ERI155-EC-IND | | *Enterobacter hormaechei subsp. xiangfangensis* strain HFB10 | 100 |
| ERI070-EC-IND | MK640790 | Firmicutes bacterium isolate ERI070-EC | | *Bacillus aryabhattai* strain S12 | 85 |
| ERI072-EC-IND | MK640791 | *Bacillus* sp. ERI072-EC-IND | | *Bacillus proteolyticus* | 99 |
| ERI108-EC-IND | MK720961 | *Pseudomonas* sp. ERI108-EC-IND | | *Pseudomonas aeruginosa* | 97 |
| ERI047-EW-IND | MK720963 | *Pseudomonas* sp. ERI047-EW-IND | | *Pseudomonas aeruginosa* | 99 |
| ERI048-EW-IND | MK640794 | *Bacillus* sp. ERI048-EW-IND | | *Bacillus haynesii* | 100 |
| ERI049-EW-IND | MK811108 | *Pseudomonas sp.* ERI049-EW-IND | | *Pseudomonas aeruginosa* | 99 |
| ERI050-EW-IND | MK720964 | *Enterobacter sp.* ERI050-EW-IND | | *Enterobacter hormaechei* subsp. xiangfangensis | 99 |
| ERI123-EW-IND | MK640795 | *Pseudomonas sp.* ERI123-EW-IND | | *Pseudomonas aeruginosa* | 99 |
| ERI056-EW-IND | MK640796 | *Pseudomonas sp.* ERI056-EW-IND | | *Pseudomonas aeruginosa* | 96 |
| ERI149-EW-IND | MK640797 | *Klebsiella sp.* ERI149-EW-IND | | *Klebsiella pneumoniae* subsp. *Rhinoscleromatis* | 99 |
| ERI150-EW-IND | MK640798 | *Enterobacter sp.* ERI150-EW-IND | | *Enterobacter bugandensis* | 99 |
| ERI151-EW-IND | MK640799 | *Bacillus sp.* ERI151-EW-IND | | *Bacillus tequilensis* | 100 |
| ERI152-EW-IND | MK640800 | *Bacillus sp.* ERI152-EW-IND | | *Bacillus nakamurai* | 100 |
| ERI153-EW-IND | MK640801 | *Enterobacter sp.* ERI153-EW-IND | | *Enterobacter mori* | 99 |
| ERI154-EW-IND | MK640802 | *Pectobacterium sp.* ERI154-EW-IND | | *Pectobacterium aroidearum* | 98 |
| ERI003-FG- | MK640803 | *Bacillus sp.* ERI003-FG- | 3$^{rd}$ | *Bacillus tropicus* | 100 |

| | | | | | |
|---|---|---|---|---|---|
| IND | | IND | instar | | |
| ERI009-FG-IND | MK640804 | *Bacillus sp*. ERI009-FG-IND | | *Bacillus cereus* | 99 |
| ERI011-FG-IND | MK640805 | *Bacillus sp*. ERI011-FG-IND | | *Bacillus nakamurai* | 100 |
| ERI080-FG-IND | MK640806 | *Bacillus sp*. ERI080-FG-IND | | *Bacillus proteolyticus* | 99 |
| ERI111-FG-IND | MK640807 | *Pseudomonas sp.* ERI111-FG-IND | | *Pseudomonas aeruginosa* | 99 |
| ERI017-FG-IND | MK640808 | *Bacillus sp*. ERI017-FG-IND | 4th instar | *Bacillus nakamurai* | 99 |
| ERI024-FG-IND | MK640809 | *Acinetobacter sp*. ERI024-FG-IND | | *Acinetobacter indicus* | 99 |
| ERI117-FG-IND | MK640810 | *Bacillus* sp. ERI117-FG-IND | | *Bacillus tequilensis* | 99 |
| ERI124-FG-IND | MK640811 | *Pseudomonas* sp. ERI124-FG-IND | | *Pseudomonas aeruginosa* | |
| ERI025-FG-IND | MK640812 | *Bacillus* sp. ERI025-FG-IND | 5th instar | *Bacillus proteolyticus* | 99 |
| ERI027-FG-IND | MK640813 | *Bacillus sp.* ERI027-FG-IND | | *Bacillus tropicus* | 99 |
| ERI038-FG-IND | MK811105 | *Pseudomonas sp.* ERI038-FG-IND | | *Pseudomonas aeruginosa* | 99 |
| ERI040-FG-IND | MK811106 | *Bacillus sp.* ERI040-FG-IND | | *Bacillus cereus* | 100 |
| ERI005-MG-IND | MK640814 | *Bacillus sp.* ERI005-MG-IND | 3rd instar | *Bacillus cereus* | 99 |
| ERI006-MG-IND | MK640815 | *Bacillus sp.* ERI006-MG-IND | | *Bacillus valezensis* | 100 |
| ERI007-MG-IND | MK640816 | *Bacillus sp*. ERI007-MG-IND | | *Bacillus tropicus* | 100 |
| ERI013-MG-IND | MK640817 | *Bacillus sp*. ERI013-MG-IND | | *Bacillus nakamurai* | 99 |
| ERI097-MG-IND | MK640818 | *Pseudomonas sp.* ERI097-MG-IND | | *Pseudomonas aeruginosa* | 99 |
| ERI110-MG-IND | MK640819 | *Bacillus sp*. ERI110-MG-IND | | *Bacillus nakamurai* | 100 |
| ERI112-MG-IND | MK640820 | *Bacillus sp*. ERI112-MG-IND | | *Bacillus nealsonii* | 99 |
| ERI019-MG- | MK640828 | *Bacillus sp.* ERI019-MG- | 4th | *Bacillus tropicus* | 99 |

| | | | | | |
|---|---|---|---|---|---|
| IND | | IND | instar | | |
| ERI020-MG-IND | MK811107 | *Bacillus sp.* ERI020-MG-IND | | *Bacillus amyloliquefaciens* | 97 |
| ERI145-MG-IND | MK640821 | *Enterobacter sp.* ERI145-MG-IND | | *Enterobacter hormaechei subsp. xiangfangensis* | 99 |
| ERI146-MG-IND | MK640822 | *Bacillus sp.* ERI146-MG-IND | | *Bacillus nakamurai* | 100 |
| ERI028-MG-IND | MK640823 | *Bacillus sp.* ERI028-MG-IND | 5th instar | *Bacillus proteolyticus* | 100 |
| ERI030-MG-IND | MK640824 | *Bacillus sp.* ERI030-MG-IND | | *Bacillus cereus* | 99 |
| ERI036-MG-IND | MK640825 | *Pseudomonas sp.* ERI036-MG-IND | | *Pseudomonas aeruginosa* | 99 |
| ERI041-MG-IND | MK720962 | *Enterobacter sp.* ERI041-MG-IND | | *Enterobacter hormaechei subsp. xiangfangensis* | 99 |
| ERI042-MG-IND | MK640826 | *Bacillus sp.* ERI042-MG-IND | | *Bacillus subtilis* | 100 |
| ERI043-MG-IND | MK640827 | *Pseudomonas sp.* ERI043-MG-IND | | *Pseudomonas aeruginosa* | 99 |
| ERI120-MG-IND | MK640829 | *Bacillus sp.* ERI120-MG-IND | | *Bacillus cereus* | 99 |
| | | | | | 99 |
| ERI113-HG-IND | MK640830 | *Bacillus sp.* ERI113-HG-IND | 3rd instar | *Bacillus subtilis subsp. inaquosorum* | |
| ERI016-HG-IND | MK640831 | *Bacillus sp.* ERI016-HG-IND | | *Bacillus haynesii* | 99 |
| ERI132-HG-IND | MK640832 | *Pseudomonas sp.* ERI132-HG-IND | | *Pseudomonas aeruginosa* | 100 |
| ERI126-HG-IND | MK640833 | *Pseudomonas sp.* ERI126-HG-IND | | *Pseudomonas mendocina* | 98 |
| ERI148-HG-IND | MK640834 | *Enterobacter sp.* ERI148-HG-IND | 4th instar | *Enterobacter hormaechei subsp. xiangfangensis* | 99 |
| ERI021-HG-IND | MK640835 | *Bacillus sp.* ERI021-HG-IND | | *Bacillus nakamurai* | 99 |
| ERI022-HG-IND | MK640836 | *Bacillus sp.* ERI022-HG-IND | | *Bacillus nakamurai* | 99 |
| ERI116-HG-IND | MK640837 | *Bacillus sp.* ERI116-HG-IND | | *Bacillus tequilensis* | 99 |
| ERI129-HG-IND | MK640838 | *Pseudomonas sp.* ERI129-HG-IND | | *Pseudomonas aeruginosa* | 99 |

| | | | | | |
|---|---|---|---|---|---|
| ERI121-HG-IND | MK640839 | *Enterococcus sp*. ERI121-HG-IND | 5th instar | *Enterococcus casseliflavus* | 100 |
| ERI044-HG-IND | MK640840 | *Bacillus sp*. ERI044-HG-IND | | *Bacillus subtilis* | 100 |
| ERI031-HG-IND | MK640841 | *Bacillus sp*. ERI031-HG-IND | | *Bacillus pacificus* | 98 |

**Supplementary Table S4.** Anaerobic gut bacteria isolated from the different compartment of *S. ricini* identified by partial 16S rRNA gene amplification

| **Sample ID** | **Gut Section** | **Closest relative in GenBank** | **Accession Number** | **Similarity (%)** |
|---|---|---|---|---|
| ES-ANE-EFG-1 | FG | *Enterobacter sp.* | MN519580 | 99 |
| ES-ANE-EFG-4 | FG | *Citrobacter freundii* | MN519581 | 96 |
| ES-ANE-EFG-5 | FG | *Pseudomonas aeruginosa* | MN519582 | 98 |
| ES-ANE-EFG-6 | FG | *Enterobacter hormaechei* | MN533929 | 99 |
| ES-ANE-EMG-2 | MG | *Enterobacter cloacae* | MN519583 | 99 |
| ES-ANE-EMG-5 | MG | *Enterococcus faecium* | MN519584 | 100 |
| ES-ANE-EMG-5-A | MG | *Enterococcus sp.* | MN519585 | 93 |
| ES-ANE-EMG-6 | MG | *Pseudomonas aeruginosa* | MN533930 | 97 |
| ES-ANE-EHG-1 | HG | *Enterobacter sp.* | MN519586 | 97 |
| ES-ANE-EHG-3 | HG | *Bacillus licheniformis* | MN519587 | 99 |
| ES-ANE-EHG-4 | HG | *Raoultella terrigena* | MN519588 | 100 |
| ES-ANE-EHG-7 | HG | *Enterobacter sp.* | MN519589 | 99 |

Key: FG=Foregut; MG=Midgut; HG=Hindgut

**Supplementary Table S5.** Diversity indices (± SE) for symbiotic gut bacteria of *S. ricini* from different gut compartments

| Section | Shannon–Wiener index (H') | Evenness (E) |
|---|---|---|
| Foregut | 1.23 ± 0.28 | 0.83± 0.03 |
| Midgut | 1.60 ± 0.38 | 0.97± 0.03 |
| Hindgut | 1.32 ± 0.15 | 0.96± 0.15 |
| Mean | 1.38 | 0.92 |
| p-Value | 0.4914 | 0.5909 |
| Tukey HSD at 5% | NS | NS |

**Supplementary Table S6. Cellulolytic and lipolytic index (Mean ± SE) of bacterial isolates from *S. ricini***

| Isolate ID | Cellulolytic Index | Lipolytic Index |
|---|---|---|
| ERI064-EC-IND | 2.33 ± 0.08 | 2.54 ± 0.60 |
| ERI155-EC-IND | 0.00 ± 0.00 | 2.67 ± 0.72 |
| ERI070-EC-IND | 0.00 ± 0.00 | 1.46 ± 0.04 |
| ERI072-EC-IND | 0.00 ± 0.00 | 1.38 ± 0.06 |
| ERI108-EC-IND | 0.00 ± 0.00 | 0.00 ± 0.00 |
| ERI047-EW-IND | 0.00 ± 0.00 | 2.63 ± 0.22 |
| ERI048-EW-IND | 0.00 ± 0.00 | 1.75 ± 0.07 |
| ERI049-EW-IND | 0.00 ± 0.00 | 2.63 ± 0.33 |
| ERI050-EW-IND | 1.51 ± 0.20 | 2.75 ± 0.52 |
| ERI123-EW-IND | 0.00 ± 0.00 | 1.42 ± 0.04 |
| ERI056-EW-IND | 0.00 ± 0.00 | 2.11 ± 0.24 |
| ERI149-EW-IND | 0.00 ± 0.00 | 1.67 ± 0.17 |
| ERI150-EW-IND | 0.00 ± 0.00 | 1.42 ± 0.17 |
| ERI151-EW-IND | 1.27 ± 0.07 | 1.33 ± 0.04 |
| ERI152-EW-IND | 2.17 ± 0.11 | 2.17 ± 0.29 |
| ERI153-EW-IND | 1.13 ± 0.00 | 1.84 ± 0.04 |
| ERI154-EW-IND | 2.04 ± 0.11 | 2.54 ± 0.04 |
| ERI003-FG-IND | 1.59 ± 0.04 | 3.05 ± 0.36 |
| ERI009-FG-IND | 1.84 ± 0.04 | 3.13 ± 0.19 |
| ERI011-FG-IND | 2.21 ± 0.18 | 1.46 ± 0.08 |
| ERI080-FG-IND | 0.00 ± 0.00 | 1.75 ± 0.07 |
| ERI111-FG-IND | 0.00 ± 0.00 | 0.00 ± 0.00 |
| ERI017-FG-IND | 2.09 ± 0.11 | 1.88 ± 0.31 |
| ERI024-FG-IND | 1.34 ± 0.04 | 2.96 ± 0.11 |
| ERI117-FG-IND | 1.29 ± 0.04 | 1.58 ± 0.11 |
| ERI124-FG-IND | 1.42 ± 0.04 | 1.42 ± 0.04 |
| ERI025-FG-IND | 1.59 ± 0.04 | 3.04 ± 0.11 |
| ERI027-FG-IND | 0.00 ± 0.00 | 1.96 ± 0.08 |
| ERI038-FG-IND | 0.00 ± 0.00 | 1.67 ± 0.18 |
| ERI040-FG-IND | 0.00 ± 0.00 | 0.00 ± 0.00 |
| ERI005-MG-IND | 1.42 ± 0.18 | 0.00 ± 0.00 |
| ERI006-MG-IND | 1.71 ± 0.04 | 1.75 ± 0.07 |
| ERI007-MG-IND | 2.84 ± 0.11 | 0.00 ± 0.00 |
| ERI013-MG-IND | 2.63 ± 0.07 | 1.50 ± 0.07 |
| ERI097-MG-IND | 2.43 ± 0.02 | 2.20 ± 0.11 |
| ERI110-MG-IND | 1.84 ± 0.11 | 1.46 ± 0.08 |
| ERI112-MG-IND | 0.00 ± 0.00 | 0.00 ± 0.00 |
| ERI019-MG-IND | 0.00 ± 0.00 | 2.08 ± 0.33 |

| | | |
|---|---|---|
| ERI020-MG-IND | 1.50 ± 0.13 | 0.00 ± 0.00 |
| ERI145-MG-IND | 0.00 ± 0.00 | 1.42 ± 0.08 |
| ERI146-MG-IND | 0.00 ± 0.00 | 0.00 ± 0.00 |
| ERI028-MG-IND | 1.42 ± 0.04 | 2.17 ± 0.40 |
| ERI030-MG-IND | 1.42 ± 0.04 | 2.88 ± 0.51 |
| ERI036-MG-IND | 1.59 ± 0.04 | 2.54 ± 0.36 |
| ERI041-MG-IND | 0.00 ± 0.00 | 0.00 ± 0.00 |
| ERI042-MG-IND | 0.00 ± 0.00 | 2.17 ± 0.00 |
| ERI043-MG-IND | 0.00 ± 0.00 | 0.00 ± 0.00 |
| ERI120-MG-IND | 1.56 ± 0.03 | 2.00 ± 0.13 |
| ERI113-HG-IND | 0.00 ± 0.00 | 1.13 ± 0.00 |
| ERI016-HG-IND | 0.00 ± 0.00 | 0.00 ± 0.00 |
| ERI132-HG-IND | 0.00 ± 0.00 | 0.00 ± 0.00 |
| ERI126-HG-IND | 0.00 ± 0.00 | 1.75 ± 0.07 |
| ERI148-HG-IND | 0.00 ± 0.00 | 0.00 ± 0.00 |
| ERI021-HG-IND | 2.29 ± 0.28 | 0.00 ± 0.00 |
| ERI022-HG-IND | 1.63 ± 0.04 | 0.00 ± 0.00 |
| ERI116-HG-IND | 0.00 ± 0.00 | 1.38 ± 0.07 |
| ERI129-HG-IND | 1.87 ± 0.01 | 0.00 ± 0.00 |
| ERI121-HG-IND | 0.00 ± 0.00 | 2.00 ± 0.07 |
| ERI044-HG-IND | 1.52 ± 0.06 | 2.21 ± 0.04 |
| ERI031-HG-IND | 1.29 ± 0.03 | 2.17 ± 0.11 |
| General Mean | 0.90 | 1.52 |
| p-Value | <.0001 | <.0001 |
| SE(d) | 0.099 | 0.316 |
| Tukey HSD at 5% | 0.4121 | 1.319 |

**Supplementary Table S7:** Results of qualitative screening for nitrate reductase activity of anaerobic gut bacteria isolates from *S. ricini*

| **Strain ID** | **Nitrate reductase activity** |
|---|---|
| ES-ANE-EFG-1 | + |
| ES-ANE-EFG-4 | + |
| ES-ANE-EFG-5 | + |
| ES-ANE-EFG-6 | + |
| ES-ANE-EMG-2 | + |
| ES-ANE-EMG-5 | - |
| ES-ANE-EMG-5-A | - |
| ES-ANE-EMG-6 | + |
| ES-ANE-EHG-1 | + |
| ES-ANE-EHG-3 | + |
| ES-ANE-EHG-4 | - |
| ES-ANE-EHG-7 | + |

Key: + = positive nitrate reductase activity; - = negative for nitrate reductase activity

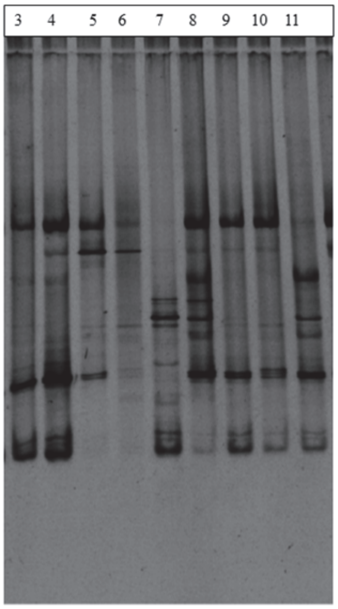


**Supplementary Figure S1**: Supplementary full-length denaturing gradient gel electrophoresis (DGGE) profile of amplified partial 16S rRNA gene at selected developmental stages of the Eri silkworm, *S. ricini.*
Key: 3=Foregut-3rd Instar, 4=Foregut-4th Instar, 5=Foregut-5th Instar, 6=Midgut-3rd Instar, 7=Midgut-4th Instar, 8=Midgut -5th Instar, 9=Hindgut-3rd Instar, 10=Hindgut-4th Instar, 11=Hindgut-5th Instar. Lanes 3 to11 have been used in Figure 2

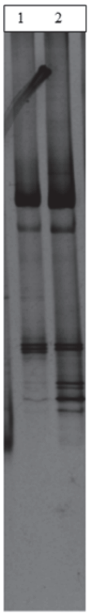


**Supplementary Figure S2:** Full-length denaturing gradient gel electrophoresis (DGGE) profile of amplified partial 16S rRNA gene bacterial isolates from the egg stage of *S. ricini.*
Key: 1=Egg wash and 2=Egg Crush homogenate samples from *S. ricini*. Lanes 1 & 2 from this figure have been used in Figure 2.

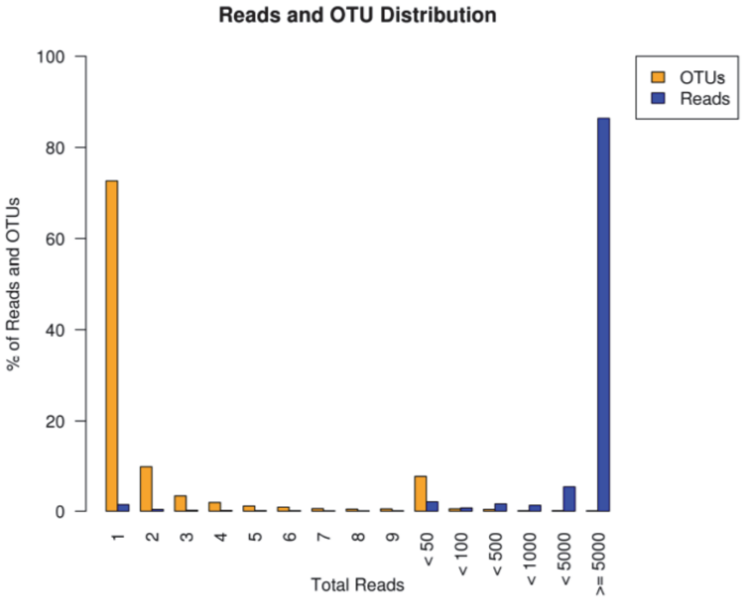


**Supplementary Figure S3**. Relative reads and OTU proportion. The blue bar represents percentage of total OTUs in the read-count groups. The orange bar represents percentage of total read contributed by the OTUs in the read-count group

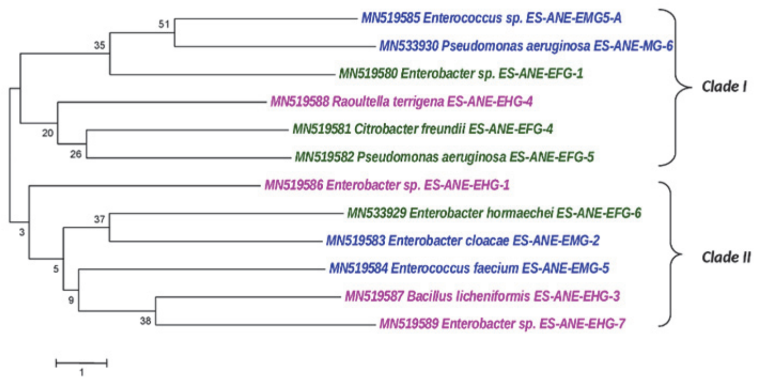


**Supplementary Figure S4.** Phylogenetic tree of facultative anaerobic gut bacteria isolated from *S. ricini*. Different colors represent the total number of bacterial colony bands that were passed on from the egg stage and persisted in 3rd, 4th and 5th instar stages of *S. ricini.* BCB represents Bacterial Colony Band.

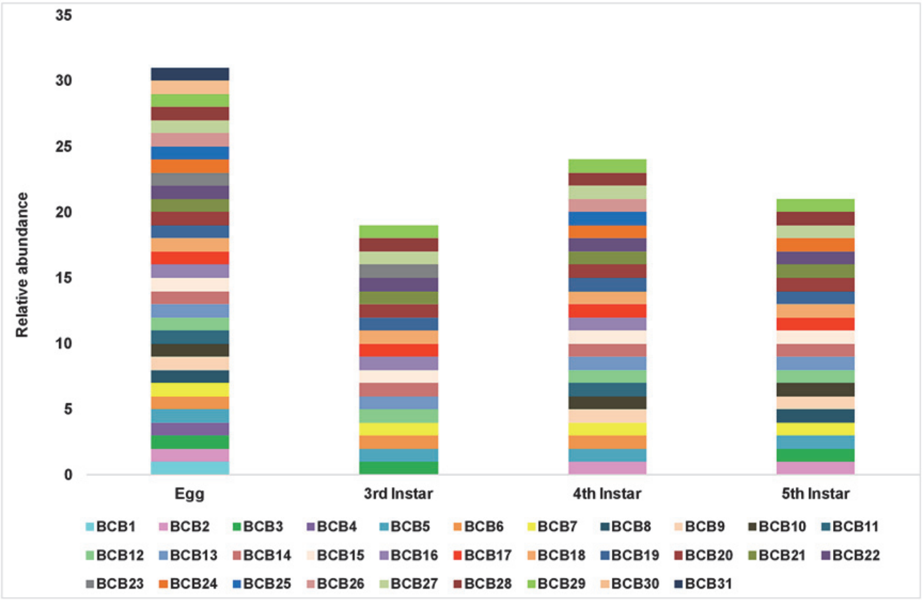


**Supplementary Figure S5.** Overview of the PCR-DGGE bacterial colony bands showing the persistence of gut bacteria across the different stages.

Different colors represent the total number of bacterial colony bands that were passed on from the egg stage and persisted in 3rd, 4th and 5th instar stages of *S. ricini.* BCB represents Bacterial Colony Band.

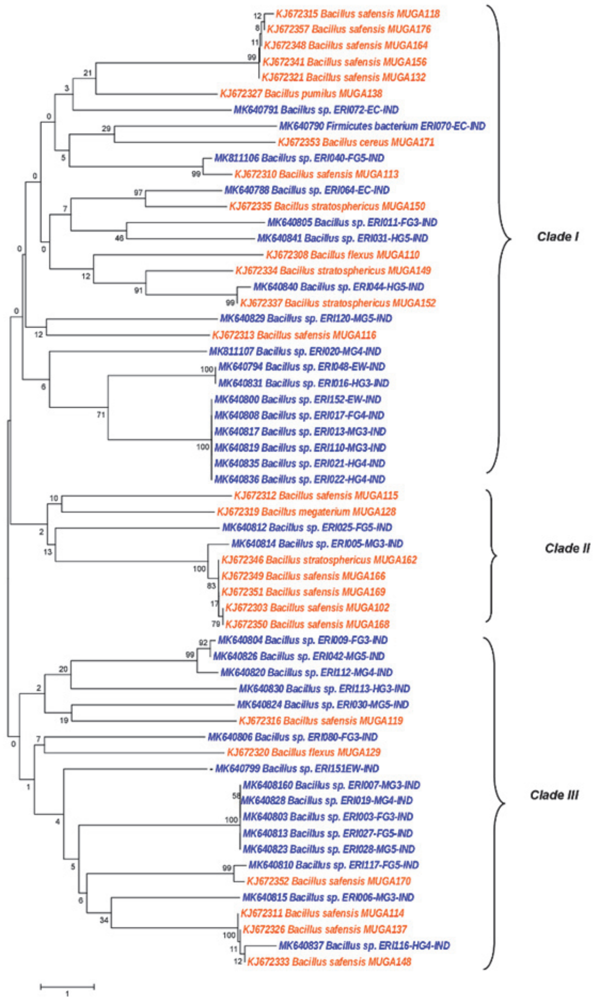


**Supplementary Figure S6.** Phylogenetic tree comparing evolutionary relationships of dominant gut bacteria isolated from two related non-mulberry sector silkworm – *Samia ricini (blue)* and *Anthereae Assamensis (pink).*
